# Motoric cognitive risk syndrome and incident hospitalization in Quebec's older population: Results of the NuAge cohort study

**DOI:** 10.3389/fmed.2022.930943

**Published:** 2022-08-16

**Authors:** Olivier Beauchet, Jacqueline Matskiv, Cyrille P. Launay, Pierrette Gaudreau, Gilles Allali

**Affiliations:** ^1^Departments of Medicine and Geriatrics, University of Montreal, Montreal, QC, Canada; ^2^Research Centre of the Geriatric University Institute of Montreal, Montreal, QC, Canada; ^3^Division of Geriatric Medicine, Department of Medicine, Sir Mortimer B. Davis Jewish General Hospital and Lady Davis Institute for Medical Research, McGill University, Montreal, QC, Canada; ^4^Lee Kong Chian School of Medicine, Nanyang Technological University, Singapore, Singapore; ^5^Research Center of the Centre Hospitalier de l'Université de Montréal, Montreal, QC, Canada; ^6^Leenaards Memory Center, Lausanne University Hospital and University of Lausanne, Lausanne, Switzerland

**Keywords:** epidemiology, cohort study, hospitalization, screening, cognitive impairment

## Abstract

**Background:**

Screening older adults at risk of hospitalization is essential to prevention of this adverse event. Motoric cognitive risk syndrome (MCR) has been associated with incident dementia and falls, which are both risk factors of hospitalization. There is no information on the association of MCR with incident hospitalization in older adults.

**Objective:**

The study aims to examine the association of MCR with incident hospitalization in community-dwelling older adults.

**Design:**

Quebec older population-based observational cohort study with 3 years of follow-up.

**Setting:**

Community dwellings.

**Subjects:**

A subset of 999 participants recruited in the NuAge study.

**Methods:**

Participants with MCR (i.e., with slow gait and cognitive complaint without dementia or motor disability) were identified at baseline assessment. Incident hospitalization (i.e., ≥1) and its recurrence (i.e., ≥2) were collected annually over a 3 year follow-up period.

**Results:**

The prevalence of MCR was 5.0% at baseline. The overall incidence of hospitalization was 29.0% and its recurrence 4.8%. MCR was associated with incident recurrent hospitalization [adjusted Hazard Ratio (aHR) = 2.58 with 95% Confidence Interval (CI) = (1.09–6.09) and *P* = 0.031], but not with incident hospitalization [aHR = 1.48, with 95%CI = (0.95–2.28) and *P* = 0.081].

**Conclusion:**

MCR is associated with incident recurrent hospitalization in NuAge participants, suggesting that MCR may be of clinical interest for screening individuals at risk for hospitalization in Quebec's older population.

## Key-points

- Slow walking speed combined with subjective cognitive complaint define motoric cognitive risk syndrome (MCR).
- MCR has been associated with incident dementia and falls, which are both risk factors for hospitalization.
- The results showed that MCR is associated with incident recurrent hospitalization in NuAge participants.
- MCR may be of clinical interest for screening community-dwelling older adults at risk of hospitalization.

## Introduction

Motoric Cognitive Risk syndrome (MCR) is a clinical syndrome associating subjective cognitive complaint with slow gait speed (1). Its worldwide prevalence is around 10% (1, 2). MCR is associated with incident adverse health outcomes in older adults including dementia, falls and mortality (1–6). MCR diagnosis is simple, rapid, low cost and thus facilitates detection of individuals at risk of adverse health outcomes in the older population (1–4).

Older adults are exposed to a greater risk of hospitalization than their younger counterparts (7–9). They are more than twice as likely to require hospitalization compared with adults in middle age (8, 9). In addition, their hospitalization is often associated with numerous adverse outcomes like long length of stay, functional decline and in-hospital death (8–10). This high risk of hospitalization and related adverse outcomes is explained in part by frailty, which is a heath condition characterized by vulnerability to stressors due to decreased physiological reserves (11, 12). Prevention of hospitalization is based on its risk screening in the older population. Assessing frailty may be a solution for the risk screening of hospitalization in older adults. Such a preventive strategy is of particular importance in COVID-19 pandemic context, as hospitals receive a high influx of patients that may exceed their capacity (13).

Cognitive frailty is defined as the simultaneous existence of both physical frailty and cognitive impairment (14). MCR may be assimilated as a cognitive frailty state, suggesting that this syndrome may be associated with increased risk of hospitalization. Furthermore, both MCR components (i.e., slow gait and subjective cognitive complaint) have been independently associated with an increased risk of hospitalization (7–10). Thus, we hypothesized that MCR could be associated with incident hospitalizations in the older population. The present study aims to examine the association of MCR and its components (i.e., slow walking speed and cognitive complaint) with incident hospitalization in community-dwelling older adults living in Quebec (Canada).

## Materials and methods

### Design and sample

**Figure 1:**
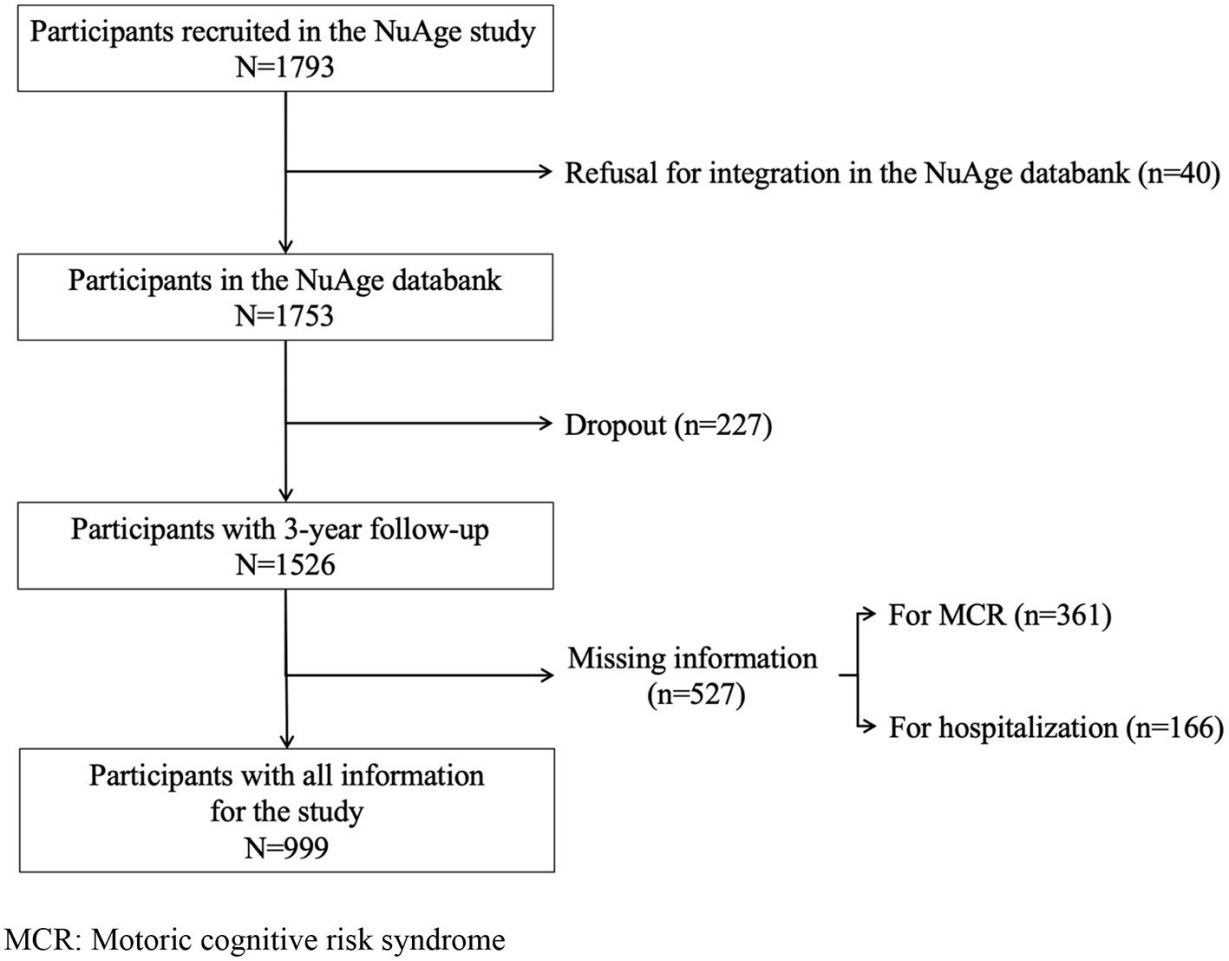
Flow diagram of the selection of the participants. MCR, Motoric cognitive risk syndrome.

The “Nutrition as a determinant of successful aging: The Quebec longitudinal study” (NuAge) study is a population-based cohort study of community-dwelling older adults carried out in Quebec (Canada), for which data about incident hospitalization was collected over a 3 year follow-up period. The present study used data from the NuAge Database and Biobank. The NuAge data collection procedure has been previously described (15). Briefly, men and women aged 67 to 84 without cognitive impairment (i.e., Modified Mini-Mental State (3MS) score >79/100) and major physical disability (i.e., able to walk 300 meters and climb 10 stairs without rest), living independently in the community and willing to commit to up to a 5 year follow-up were enrolled (16). A total of 1,793 participants were recruited between November 2003 and June 2005. Among them, 1,753 (97.8%) agreed to the integration of their data and biosamples into the NuAge Database and Biobank for future studies. From this subset, 1,526 (85.1%) were followed over a 3 year period. We excluded participants with missing values for MCR and hospitalization. Finally, 999 (57.0%) participants from the original set were selected for the present study. A flow diagram illustrating the selection of participants is shown in the Figure 1.

### Assessment

Age, sex, living alone, place of living (individual home *vs*. residence), measured weight (kg) and height (cm), and number of medications taken daily were recorded at baseline. Overweight or obesity was defined as body mass index (BMI) ≥ 25 kg/m^2^ and underweight <18.5 kg/m^2^. Frailty state was assessed using the CARE scale (17). CARE is a validated scale composed of 21 items counting health deficits (i.e., symptoms, signs, diseases and disability) and age and sex, as described previously (17). CARE is based on the idea that a greater number of deficits indicates a higher frailty state (18, 19). Its score ranges from 0 (no deficit) to 21 (all deficits present) and its stratification separates individuals in robust (score 0–1), pre-frail (score 2–4) and frail (score ≥5) states (please see the Supplementary material for more details).

### Definition of motoric cognitive risk syndrome at baseline

MCR was defined using information collected at baseline as a combination of subjective cognitive complaint (SCC) and slow walking speed in the absence of dementia and gait disability (1). Subjective cognitive complaint was defined as the following: a “yes” response to the question “*Do you feel you have more problems with memory than most*?” from the 30-item Geriatric Depression Scale (GDS) and/or as impairment in memory recorded using the memory item of Functional Autonomy Measurement System (SMAF) (20, 21). Walking speed (m/s) was measured using a standardized procedure. Participants were asked to walk a 4-meter distance at their usual pace twice. The time (in second) was recorded between the second and the fourth meter. The best of the two attempts was used for this study. Slow walking speed was defined as a walking speed at least one standard deviation (SD) below the age-appropriate mean values established in the present cohort. Participants were divided into two sex groups and four age groups, as described by Verghese et al. (1, 2). The cut-off scores for defining slow gait were <1.09 m/s for males in age group 67–72, <1.00 m/s for the age group 73–77, <0.97 for the age group 78–84 and <0.93 m/s for the age group ≥ 85; and they were <1.04 m/s for females in the age group 67–72, <0.97 m/s for the age group 73–77, <0.91 m/s for the age group 78–84 and <0.81 cm/s for the age group ≥ 85.

### Follow-up

The follow-up period was 3 years. Information about the number of hospitalizations over the past year was collected annually. Participants were separated into three groups: No incident hospitalization, at least 1 incident hospitalization over the 3 years (i.e., incident hospitalization) and at least two hospitalizations over the 3 years (i.e., incident recurrent hospitalizations). The last follow-up was performed in June 2008.

### Standard protocol approval and patient consents

The NuAge protocol was approved by the Research Ethics Boards (REB) of the University Institute of Geriatrics of Sherbrooke and the “*Institut universitaire de gériatrie de Montréal*.” Written informed consent for research was obtained for all recruited NuAge participants. The REB of the CIUSSS-de-l'Estrie-CHUS approved the NuAge Database and Biobank. The present study was approved by the REB of the Jewish General Hospital (Montreal, Quebec, Canada). The NuAge data set used in this study was transmitted by the NuAge Database team on May 07, 2019.

### Statistics

The participants' baseline characteristics were described with means, Standard Deviation (SD), and percentages. Participants were separated into two groups based on their MCR status (i.e., with and without MCR). First, comparisons between groups were performed using unpaired *t*-tests or Chi-squared tests. Second, Cox regressions were performed to examine the association of MCR and its components (i.e., slow walking speed and cognitive complaint) used as independent variables (separated model for each variable) with incident hospitalizations, which were labeled as at least one hospitalization or two hospitalizations (dependent variable with separated models for each type of hospitalization). Unadjusted and adjusted models by frailty were examined. Frailty was defined as CARE score ≥ 2 (i.e., pre-frail and frail participants pooled together). *P*-values < 0.05 were considered statistically significant. All statistics were performed using SPSS (version 24.0; SPSS, Inc., Chicago, IL).

## Results

Table 1 compares the baseline characteristics of participants with and without MCR. The prevalence of MCR was 5% at baseline and the overall incidence of hospitalizations (i.e., ≥1) was 29.0% and its recurrence (i.e., ≥2) 4.8%. MCR participants lived less frequently at their individual home (*P* = 0.002) and had polypharmacy more frequently (*P* = 0.038) compared to those without MCR. There were also significantly more pre-frail and frail participants in the MCR group (*P* ≤ 0.001). Both hospitalization (*P* = 0.017) and recurrent hospitalizations (*P* = 0.015) were more incident in the MCR participants compared to the non-MCR participants. MCR participants had slower walking speed and higher memory complaint compared to non-MRC counterparts (*P* ≤ 0.001). Unadjusted Cox regressions revealed that MCR [Hazard ratio (HR) ≥ 1.56 with P ≤ 0.046; Table 2] and slow walking speed (HR ≥ 1.40 with *P* ≤ 0.021) were associated with hospitalizations, regardless of their recurrence. Adjustment by frail stage led to a non-significant association between MCR and at least one incident hospitalization (HR = 1, 48 with *P* = 0.081), whereas the association with incident recurrent hospitalizations remained significant (HR = 2.58 with *P* = 0.031). Slow walking speed was still associated with hospitalizations, regardless of their recurrence (HR ≥ 1.37 with *P* ≤ 0.028). No association between cognitive complaint and hospitalization was found. Frailty stage was associated with at least one incident hospitalization in adjusted models (HR ≥ 1.84 with *P* ≤ 0.016), but not with recurrent hospitalizations.

**Table 1:** Baseline characteristics of the NuAge participants stratified by their motoric cognitive risk syndrome status (*n* = 999).

	**MCR participants**		***P*** -Value^*^
	**No** **(*n* = 949)**	**Yes** **(*n* = 50)**	
**Age (years)**			
Mean ± SD	73.9 ± 4.1	74.8 ± 4.0	0.136
>80, *n* (%)	111 (11.7)	7 (14.0)	0.623
Female, *n* (%)	506 (53.3)	23 (46.0)	0.312
Living alone, *n* (%)	300 (31.6)	15 (30.0)	0.811
Place of living individual home, *n* (%)	899 (94.7)	42 (84.0)	**0.002**
Body mass index abnormal^†^, *n* (%)	689 (72.6)	41 (82.0)	0.144
Polypharmacy^‡^, *n* (%)	427 (45.0)	30 (60.0)	**0.038**
**CARE Frailty state^¶^**, ***n*** **(%)**			
Robust	106 (11.2)	-	-
Pre-frail	666 (70.2)	21 (42.0)	**≤0.001**
Frail	172 (18.1)	29 (58.0)	**≤0.001**
**Walking speed, mean ±SD (m/s)**			
Mean ± SD (m/s)	1.16 ± 0.19	0.86 ± 0.11	**≤0.001**
Slow gait speed^#^, *n* (%)	113 (11.9)	50 (100)	**≤0.001**
Cognitive complaint^**^, *n* (%)	188 (19.8)	50 (100)	**≤0.001**
**Incident hospitalization**, ***n*** **(%)**			
≥1	268 (28.2)	22 (44.0)	**0.017**
≥2	42 (4.5)	6 (12.0)	**0.015**

*MCR, Motoric cognitive risk syndrome; SD, Standard deviation;*

* *Based on unpaired t-test or Chi-Square test, as appropriate;*

† *≥25 or <18.5 kg/m^2^;*

‡ *Number of drugs daily taken ≥5;*

¶ *Frailty scale with score ranged between 0 (no frailty) and 21 (highest frailty) and stratification in three levels including robust (0-1), pre-frail (2–4) and frail (≥5);*

# *Defined as a walking speed at least one standard deviation (SD) below the age-appropriate mean values established in the present cohort;*

** *Defined as the following: a “yes” response to the question “Do you feel you have more problems with memory than most?” from the 30-item Geriatric Depression Scale (GDS) and/or as impairment in memory recorded using the memory item of Functional Autonomy Measurement System; P-Value significant (i.e., <0.05) indicated in bold*.

**Table 2:** Cox regressions showing the association of hospitalizations (dependent variable) with motoric cognitive risk syndrome (independent variable) (*n* = 999).

	**Model 1**						**Model 2**					
	**Hospitalization ≥ 1**			**Hospitalization ≥ 2**			**Hospitalization ≥ 1**			**Hospitalization ≥ 2**		
	**HR**	**[95%CI]**	***P*** **-Value**	**HR**	**[95%CI]**	***P*** **-Value**	**HR**	**[95%CI]**	***P*** **-Value**	**HR**	**[95%CI]**	***P*** **-Value**
Cognitive complaint^*^	1.24	[0.96; 1.60]	0.103	1.19	[0.63; 2.4]	0.599	1.17	[0.90; 1.51]	0.243	1.12	[0.59; 2.13]	0.735
Frailty^†^		-			-		1.84	[1.12; 3.01]	**0.016**	1.74	[0.54; 5.67]	0.356
Slow walking speed^‡^	1.40	[1.05; 1.84]	**0.021**	2.33	[1.27; 4.29]	**0.007**	1.37	[1.04; 1.82]	**0.028**	2.29	[1.24; 4.22]	**0.008**
Frailty^†^		-			-		1.87	[1.14; 3.05]	**0.013**	1.67	[0.52; 5.37]	0.393
MCR	1.56	[1.01; 2.40]	**0.046**	2.71	[1.15; 6.37]	**0.022**	1.48	[0.95; 2.28]	0.081	2.58	[1.09; 6.09]	**0.031**
Frailty^†^		-			-		1.86	[1.14; 3.04]	**0.013**	1.64	[0.51; 5.32]	0.406

*MCR, motoric cognitive risk syndrome; HR, Hazard ratio; Model 1, Unadjusted model; Model 2, Adjusted by frail state (i.e., Pre-frail and frail individuals were pooled together as the group used as independent covariate); CI: Confident interval;*

* *: Defined as the following: a “yes” response to the question “Do you feel you have more problems with memory than most?” from the 30-item Geriatric Depression Scale (GDS) and/or as impairment in memory recorded using the memory item of Functional Autonomy Measurement System;*

† *: Defined as CARE score ≥ 2;*

‡ *: Defined as a walking speed at least one standard deviation (SD) below the age-appropriate mean values established in the present cohort; P-Value significant (i.e., <0.05) indicated in bold*.

## Discussion

Our findings show that MCR and one of its components, which is slow walking speed, are significantly associated with incident recurrent hospitalizations in Nuage participants, independently of their frail state. Furthermore, the greatest association was found with MCR status.

To the best of our knowledge, it is the first time that an association between MCR status and the occurrence of hospitalizations is being reported. The slow walking speed component of MCR exposes the individual to a greater risk for falls (22). In Canada, falls are a significant leading cause of hospitalizations, which may in part explain the association between MCR and hospitalization^1^. Furthermore, it has been reported that many patients who visit the emergency department are more prone to hospital admission (8–10). Those most at risk of hospitalization are patients with cognitive impairment such as dementia (9, 23). MCR is a pre-dementia stage. Therefore, it may be suggested that patients at the onset of dementia may be more prone to hospitalization. Finally, it was reported that the risk of dementia was greater for MCR compared to its components alone, due to a synergistic effect (24). We reported the same results, the greatest association being between MCR and recurrent hospitalizations compared to each MCR component respectively. Therefore, the same effect could be postulated for the risk of hospitalization in individuals with MCR. This result highlights the benefit of using MCR to screen for hospitalization of older adults.

Our findings also revealed that the association of MCR with incident recurrent hospitalization was independent of frail state. Frailty is as an individual's health state as characteriazed by vulnerability to stressors due to decreased physiological reserves (11, 12). Frailty exposes the individual to a greater risk of hospitalization (17–19). It may suggested that MCR identified frail individuals. Indeed, MCR may be assimilated into a “cognitive frailty” state which is a clinical condition with co-existing physical frailty and cognitive impairment in non-demented older adults (14). Slow walking is a sign of physical frailty-which may explain in part its association with hospitalization regardless of its recurrence-while subjective cognitive impairment is the first stage of cognitive impairment (1–4). Furthermore, our findings showed that MCR is associated with recurrent hospitalizations, as a new adverse outcome, in addition to those previously reported in the literature, which are dementia, falls and mortality (2–4). Because frailty is a health state that exposes individuals to a greater risk of adverse outcomes, MCR seems to meet all the criteria for the identification of frail individuals. Finally, the fact that a significant association between MCR and incident hospitalizations was found only with recurrent hospitalizations reinforces this proposition. Indeed, recurrent hospitalizations are particularly incident in frailer patients (17, 18). It has been shown that older adults with recurrent hospitalizations are individuals with multi-morbidities and a high need for care, with increasingly unmet needs (25, 26). Thus, recurrent hospitalizations may be posited to result from complex interactions between patients' physical and mental conditions, social situation, and issues related to the provision of care.

The NuAge sample size and its follow-up period duration are both strengths of the present study. However, some limitations need to be considered. First, even if the prospective and observational design was appropriate to the objective of our study, examining an association between MCR and incident hospitalization was not initially planned. Second, we selected 56.9% of the initial participants recruited in the NuAge study, which may not adequately represent the whole cohort. Third, the generalizability of the findings may be limited because data were collected 15 years ago and older adult characteristics have changed significantly over the past decade. Additionally, the COVID-19 pandemic has likely contributed to changes in the distribution of frailty in Quebec's older population.

In conclusion, MCR is associated with incident recurrent hospitalizations in participants of the NuAge cohort study, suggesting that MCR may be of clinical interest when screening individuals at risk for hospitalization in the Quebec older adult population.

## Data availability statement

The data analyzed in this study is subject to the following licenses/restrictions: Access to the NuAge Database can be obtained by completing on access request on their website: https://nuage.recherche.usherbrooke.ca/en/faire-une-demande-dacces. Requests to access these datasets should be directed to https://nuage.recherche.usherbrooke.ca/en/faire-une-demande-dacces.

## Ethics statement

The studies involving human participants were reviewed and approved by REB of the Jewish General Hospital (Montreal, Quebec, Canada). The patients/participants provided their written informed consent to participate in this study.

## Author contributions

Conceived and designed the experiments and analyzed and interpreted the data: OB and GA. Cohort data collection: PG. Contributed reagents, materials, and analysis tools or data: OB. Writing of the manuscript: OB, JM, and GA. Revision of manuscript: CL and PG. All authors contributed to the article and approved the submitted version.

## Funding

The NuAge Study was funded by the Canadian Institutes of Health Research (CIHR; MOP-62842). The NuAge Database and Biobank are supported by the Fonds de recherche du Québec (FRQ; 2020-VICO-279753), the Quebec Network for Research on Aging, a thematic network funded by the FRQ-Santé, and by the Merck-Frosst Chair funded by La Fondation de l'Université de Sherbrooke. An access to this research bank can be asked by completing an access request on their website (https://nuage.recherche.usherbrooke.ca/en/). OB and GA were supported by the National Institute of Health/National Institute on Aging grants PO1 AG03949 and R01AG057548-01A1. The French Ministry of Health financially supported the study. The sponsor had no role in designing and conducting the study, nor in the collection, management, analysis, and interpretation of the data, nor in the preparation, review, or approval of the manuscript and writing of the study.

## Conflict of interest

The authors declare that the research was conducted in the absence of any commercial or financial relationships that could be construed as a potential conflict of interest.

## Publisher's note

All claims expressed in this article are solely those of the authors and do not necessarily represent those of their affiliated organizations, or those of the publisher, the editors and the reviewers. Any product that may be evaluated in this article, or claim that may be made by its manufacturer, is not guaranteed or endorsed by the publisher.

## Footnotes

^1^ https://www.canada.ca/en/public-health/services/reports-publications/health-promotion-chronic-disease-prevention-canada-research-policy-practice/vol-40-no-9-2020/injury-hospitalizations-canada-2018-2019.html
