# Supplementary material for: Motoric cognitive risk syndrome and incident hospitalization in Quebec's older population: Results of the NuAge cohort study
Source: Front Med (Lausanne). 2022 Aug 16;9:930943. doi: 10.3389/fmed.2022.930943 (PMC9424658; doi:10.3389/fmed.2022.930943)
Supplement: Supplementary file 1 [file Table_1.pdf]

Table. CARE items value in the NuAge participants (n=999)

Characteristic	Value	[95%CI]
**CARE items**		
Age ≥ 80, n (%)	118 (11.8)	[10.5-14.9]
Male, n (%)	470 (47.0)	[43.8-53.4]
Body mass index abnormal*, n (%)	730 (73.1)	[65.9-76.5]
No vitamin D Supplementation, n (%)	252 (25.2)	[23.9-29.7]
Polypharmacy†, n (%)	457 (45.7)	[44.7-54.0]
Unable to stand up from chair five-time, n (%)	13 (1.3)	[0.5-1.7]
Use of walking aid‖, n (%)	34 (3.4)	[2.6-5.1]
Housebound‡, n (%)	1 (0.1)	[0.0-0.3]
Memory issue, n (%)	193 (19.3)	[18.9-24.3]
Feeling of empty life¶, n (%)	182 (18.2)	[17.3-22.6]
Caregiver#, n (%)	194 (19.4)	[18.4-23.7]
Social isolationα, n (%)	18 (1.8)	[1.1-3.0]
Need help for, n (%):		
Phone use[∞]	34 (3.4)	[2.6-5.1]
Transportation[µ]	8 (0.8)	[0.1-0.9]
Medication intake**	23 (2.3)	[0.2-3.6]
Handle finances††	112 (11.2)	[10.2-14.6]
Bathing	15 (1.5)	[0.1-2.4]
Toileting	122 (12.2)	[11.4-15.9]
Dressing	8 (0.8)	[0.0-1.5]
Feeding	2 (0.2)	[0.0-0.5]
Incontinence[‖‖]< n (%)	292 (29.2)	[28.7-34.8]

**CARE frailty stratification**¶¶:		
Robust, n (%)	106 (10.6)	[9.1-12.6]
Pre-Frail, n (%)	687 (68.8)	[66.2-72.0]
Frail, n (%)	201 (20.1)	[17.7-22.7]

SD: standard deviation; CI: confident interval; CHS: Cardiovascular Health Study

*: >24.9 or <18.5 kg/m^2

†: Number of drugs daily taken ≥5

‡: Related to bad health condition

||: Regardless of the type of walking aid

¶: Answer to the 30-item Geriatric Depression scale "*do you feel that your life is empty*?" yes

#: To be an individual who gives regularly care to another who need help taking care of her/himself

α: Living alone, had no home help and no contact with another person over the past week

∞: Answers telephone and dials few memorized numbers OR Does not dial OR Does not use telephone at all

µ: Travels on public transportation when accompanied by another OR Travel limited to taxi or automobile with assistance of another OR Does not travel at all

**: Need weekly supervision OR takes responsibility if medication is prepared in advance in separate dosage OR Is not capable of dispensing own medication

††: Manages day-to-day purchases, but needs help with certain major purchases OR for regular purchases OR Incapable of handling money

||||: Partially or totally incontinent of bowel or bladder

¶¶: Score ranged between 0 (no frailty) and 21(highest frailty) with three levels: robust (0-1), pre-frail (2-4) and frail (≥5).
